# OrthoDB v10: sampling the diversity of animal, plant, fungal, protist, bacterial and viral genomes for evolutionary and functional annotations of orthologs

**DOI:** 10.1093/nar/gky1053

**Published:** 2018-11-05

**Authors:** Evgenia V Kriventseva, Dmitry Kuznetsov, Fredrik Tegenfeldt, Mosè Manni, Renata Dias, Felipe A Simão, Evgeny M Zdobnov

**Affiliations:** 1 Department of Genetic Medicine and Development, University of Geneva Medical School, rue Michel-Servet 1, 1211 Geneva, Switzerland; 2 Swiss Institute of Bioinformatics, rue Michel-Servet 1, 1211 Geneva, Switzerland

## Abstract

OrthoDB (https://www.orthodb.org) provides evolutionary and functional annotations of orthologs. This update features a major scaling up of the resource coverage, sampling the genomic diversity of 1271 eukaryotes, 6013 prokaryotes and 6488 viruses. These include putative orthologs among 448 metazoan, 117 plant, 549 fungal, 148 protist, 5609 bacterial, and 404 archaeal genomes, picking up the best sequenced and annotated representatives for each species or operational taxonomic unit. OrthoDB relies on a concept of hierarchy of levels-of-orthology to enable more finely resolved gene orthologies for more closely related species. Since orthologs are the most likely candidates to retain functions of their ancestor gene, OrthoDB is aimed at narrowing down hypotheses about gene functions and enabling comparative evolutionary studies. Optional registered-user sessions allow on-line BUSCO assessments of gene set completeness and mapping of the uploaded data to OrthoDB to enable further interactive exploration of related annotations and generation of comparative charts. The accelerating expansion of genomics data continues to add valuable information, and OrthoDB strives to provide orthologs from the broadest coverage of species, as well as to extensively collate available functional annotations and to compute evolutionary annotations. The data can be browsed online, downloaded or assessed via REST API or SPARQL RDF compatible with both UniProt and Ensembl.

## INTRODUCTION

Genomic sequencing is the most comprehensive method for the molecular interrogation of organisms, with the potential to reveal the complete repertoire of genes and enable the study of cellular processes at the molecular level. Homology, the recognition of gene sequence similarities as evidence of shared ancestry, allows for hypotheses on a gene’s function when biological roles of related genes in other species are characterized. Homologs with a reference to a specific phylogeny radiation, i.e. descendants from a single gene of the last common ancestor, are termed orthologs and referred to below as ortholog groups or OGs (1,2). Such gene genealogies, pinned to particular ancestor genes, enable the most specific functional hypothesis for the descendant genes (3,4). Orthology is also the cornerstone for comparative evolutionary studies. The large-scale delineation of gene orthology is a popular but challenging task as evidenced by numerous proposed approaches (5–14).

OrthoDB is one of the largest resources of orthologs (15). Beyond the benchmarks of the underlying algorithm (15,16), the accuracy of our methodology earned its reputation through many comparative genomic studies (e.g. 17–19), particularly in the i5K initiative (20). The concept of orthologous groups is inherently hierarchical, as each phylogenetic clade or subclade of species has a distinct common ancestor; OrthoDB has explicitly emphasized this aspect since its inception (21). The ortholog delineation procedure is applied at each major radiation of the species taxonomy to produce more finely resolved groups of closely related species and to allow users to select the most relevant level.

**Figure 1.**
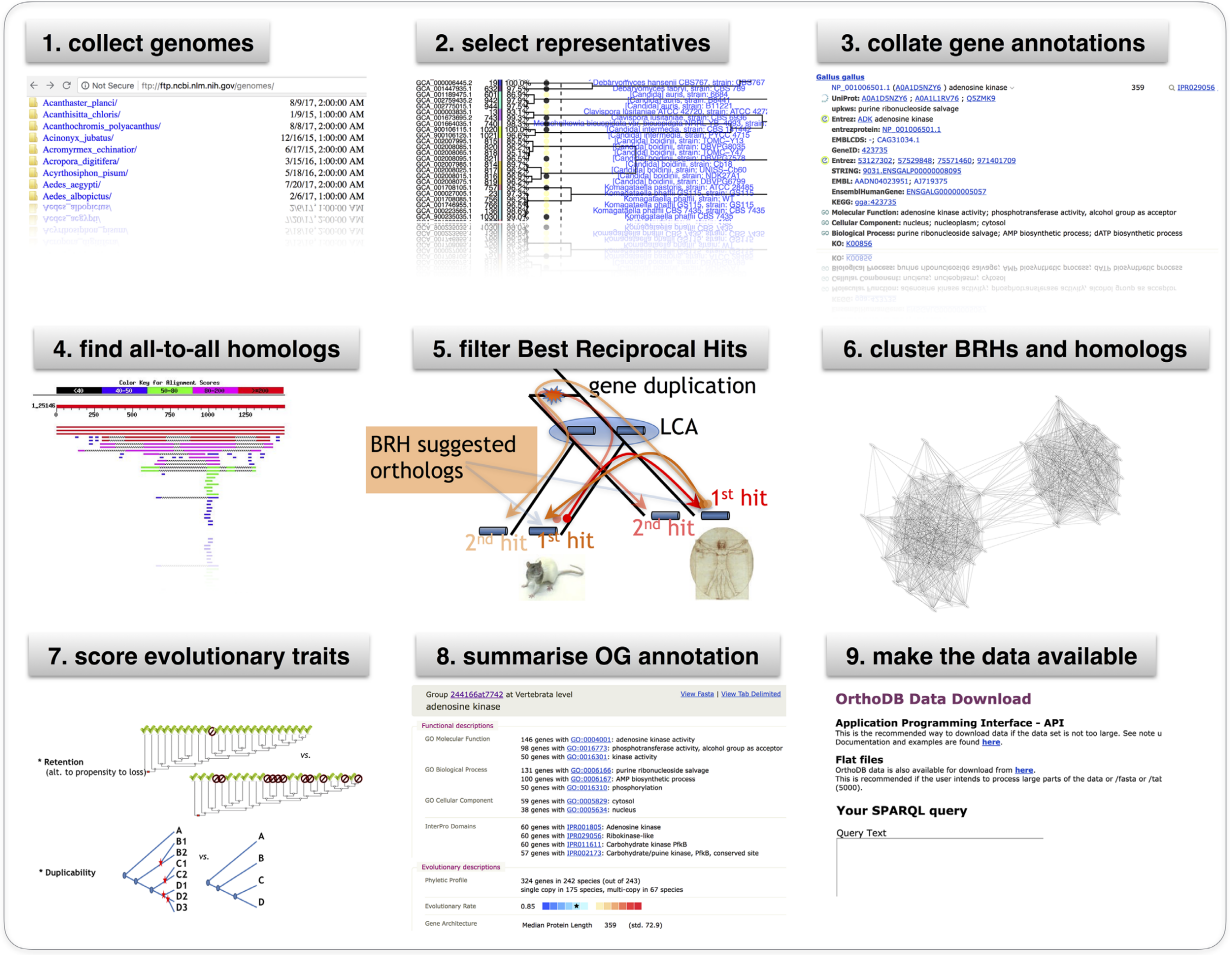
OrthoDB graphical abstract depicting the data processing pipeline.

OrthoDB provides tentative functional annotations of groups of orthologs and mapping to functional categories by summarizing functional gene annotations, extensively collected from other public resources. Annotation of genes is complicated and contains errors. Although in many cases OrthoDB makes such errors in the underlying data apparent, discordant annotations should be considered with caution. The evolutionary annotations of the orthologs remain another distinguishing feature of OrthoDB (Figure 1). In this update (v10) we further increased the coverage of organisms, adjusted the underlying algorithm, and improved usability of the web interface.

## COVERAGE OF ORGANISMS

A substantial fraction of widely inherited genes evolve under the single-copy control (22). These are the easiest to predict and are the basis for our BUSCO tool (23). The BUSCO acronym stands for Benchmarking Universal Single-Copy Orthologs. The software aims at quantitative assessment of completeness of genome assemblies, gene sets or transcriptomes, based on evolutionarily informed expectations of gene content, complementing the technical metrics like N50. Although we derive these BUSCO marker genes from OrthoDB, OrthoDB also strives to resolve the challenging cases of gene duplications and losses. Sampling of species diversity was shown to be a major factor affecting accuracy of inferred gene orthology, besides the quality of the underlying genomes and their annotations (16). OrthoDB thus strives to cover as much sequence diversity as practical with our computational resources. When there are multiple genomes available with greater than about 96% identity using MASH estimates (24), we sample the best annotated representatives with the most complete gene sets according to BUSCO metrics (25).

OrthoDB v10 now covers 1271 eukaryotes, 5609 bacteria, 404 archaea and 6488 viruses, as detailed in Table 1 (with the figures from the other resources as of September 2018). Overall, OrthoDB v10 covers over 37 million genes, classifying them into over 8.5 million tentative groups of orthologs at 624 levels of granularity. The orthology-levels, referring to the last common ancestors from which extant orthologs evolved, are defined according to the NCBI Taxonomy (26). Protein-coding gene translations for this release were retrieved mostly from RefSeq and NCBI complete genomes and the genome assembly ID is referenced now in the browsable taxonomy of organisms.

**Table 1.** Coverage of genomic diversity by the largest orthology resources

	OrthoDB	KEGG-OC	eggNOG	OMA
Eukaryota	1271	394	238	383
- Metazoa	448	n.a.	89	156
— Vertebrata	243	n.a.	51	71
— Arthropoda	170	n.a.	22	52
- Viridiplantae	117	n.a.	23	54
- Fungi	549	n.a.	85	107
Bacteria	5609	4301	1678^a^	1635
Archaea	404	253	115	149
Viruses	6488	0	352	0

^a^Plus 1655 additional bacteria were subsequently mapped.

## THE ALGORITHM AND SOFTWARE

The OrthoDB computational pipeline for delineation of orthologs is based on assessments of pairwise gene homology between complete genomes and their subsequent clustering (Figure 1). The pipeline has previously been described (15), and our software is freely available from https://orthodb.org/software.

The latest adjustments to the OrthoDB algorithm include: (i) optimizations of the underlying data structures to reduce the memory usage, enabling us to increase the number of organisms covered, (ii) use of MMseqs2 (27) for homology searches to speed up the computations growing as square of the total number of genes, (iii) introduction of an additional species overlap criteria (6) for merging seed clusters to improve accuracy of inferred orthology and (iv) splitting of frequently mispredicted gene fusions to avoid spurious association of different orthologs.

## FUNCTIONAL AND EVOLUTIONARY ANNOTATIONS

*Gene functional annotations* were collated from the major resources including Uniprot and NCBI gene records, as well as InterPro and Gene Ontology (GO). All data were processed to assemble consolidated and non-redundant per-gene annotation records, presented as short one-line descriptions that are click-expandable to immediately access the complete annotation record. The relative amount of available annotation data per gene is indicated by the size (one to five chevrons) of the click-expandable widget. Notably, we collated and made searchable references such as to KEGG pathways and to Online Mendelian Inheritance in Man (OMIM^®^) linking to human diseases.

*OG functional annotations* are consequent aggregations from their corresponding gene-level annotations, aiming to provide the user with an overview of the possible functions of the member orthologs. The compilation of one-line orthologous group descriptors to briefly but precisely outline functional knowledge in a human-readable language is a non-trivial task. We achieved this by identifying the best scoring single phrase found in any part of available annotation for all genes in the orthologous group. All these phrases were matched using a full-text search engine educated with a list of biological stop words, against a body of all annotation records of all genes in the group. This querying was performed separately for subsets of the annotations, partitioned according to data provenance. An empirically evaluated weight factor was used to choose the best phrases from each source, with the data source precedence as follows: Uniprot to ENSEMBL to NCBI to Interpro and then GO.

*Functional categories* of COG, GO, and KEGG pathways were assigned to OGs whenever possible. Such high-level functional descriptors are informative for comparative studies, e.g. in metagenomics.

*Evolutionary annotations* are computed for each OG from the available genomic data and sequence alignment scores. As detailed earlier (15), these metrics include: ‘*phyletic profile’* that reflects gene universality (proportion of species with orthologs) and duplicability (proportion of multi-copy versus single-copy orthologs), ‘*evolutionary rate’* that reflects the relative constraints on protein sequence conservation or divergence and ‘*sibling groups’* that reflects the sequence uniqueness of the orthologs. The universality of a gene family hints on a function that is widely necessary and basal, for example Orco is an essential co-receptor for insect odorant-sensing, while a lineage-restricted genes may underlie the lineage-specific adaptations, for example the *Drosophila* gland-specific peptide 26Ab. Duplicability is also indicative of the type of molecular functions, e.g. members of a signal-transduction pathway or a protein complex may evolve under the single-copy control (22), both of the examples above show this pattern (and the handful of ‘duplications’ or ‘missing’ of Orco actually point to deficiencies of the underlying genomes or their annotations). ‘*Evolutionary rate’* is a relative measure, where slower than genome average evolution may indicate stronger purifying selection, like in the case of the Orco gene, while the faster than average evolutionary rate may indicate positive selection, like in the case of the gland-specific peptide 26Ab. Although specific function of the 26Ab peptide is still unknown, this protein is transferred from male to female during mating and may act as key player in species-specific male reproductive success and thus, may be subject to rapid evolution resulting from sexual conflict and competition. The ‘*sibling groups’* allow navigation to gene families possibly having similar molecular functions. These annotations providing an evolutionary perspective remain unique to OrthoDB.

## WEB INTERFACE

The OrthoDB resource is public. The optional registration allows the authenticated users to upload their own data for performing online BUSCO analysis and for mapping to current OrthoDB OGs, enabling the user to explore mapped functional annotations and to generate user-tailored comparative charts depicting the total gene count, the fraction of common genes, the fraction of the most conserved single-copy genes, etc. The growing number of available genomes may hamper the user experience while browsing the data. Therefore, by default, orthologs from only user-selected or reference species are shown, and users may choose to toggle a check-box to view orthologs from all available species.

## CONCLUSION AND PERSPECTIVES

The growing number of sequenced genomes increases the power of comparative analyses, but it also presents challenges regarding scalability of methods and data presentation to end users. OrthoDB strives to informatively sample the available genomic space and to refine the accuracy of ortholog delineations.

## DATA AVAILABILITY

As for the previous versions of OrthoDB we provide data files for bulk download, one file per level of orthology; as well as the underlying amino acid gene translations. To retrieve substantial subsets of data from OrthoDB or to access it programmatically we provide a REST API, documented at https://www.orthodb.org/v10/?page=api, that returns data in *JSON, FASTA* or *TAB* formats. All data are distributed under the Creative Commons Attribution 3.0 License from https://www.orthodb.org/.

The RDF SPARQL interface was introduced in the previous OrthoDB v9.1 and it is gaining momentum, being compatible with both UniProt and Ensembl SPARQL endpoints. Adopting Uniform Resource Identifier (URI) of UniProt proteins and Ensembl genes, it provides the possibility for very elaborate queries and a number of clickable links to Ensembl Genomes, NCBI, Interpro and GO resources.

Users can also navigate to OrthoDB records by following links from FlyBase ‘Orthologs’ section, UniProt ‘Phylogenomic databases’ section or NCBI ‘Additional links/ Gene LinkOut’ section.

## References

[1] Fitch W.M. Homology a personal view on some of the problems. Trends Genet. 2000; 16:227–231. 10782117 10.1016/s0168-9525(00)02005-9

[2] Koonin E.V. Orthologs, paralogs, and evolutionary genomics. Annu. Rev. Genet. 2005; 39:309–338. 16285863 10.1146/annurev.genet.39.073003.114725

[3] Tatusov R.L., Koonin E.V., Lipman D.J. A genomic perspective on protein families. Science. 1997; 278:631–637. 9381173 10.1126/science.278.5338.631

[4] Gabaldon T., Koonin E.V. Functional and evolutionary implications of gene orthology. Nat. Rev. Genet. 2013; 14:360–366. 23552219 10.1038/nrg3456 PMC5877793

[5] van der Heijden R.T., Snel B., van Noort V., Huynen M.A. Orthology prediction at scalable resolution by phylogenetic tree analysis. BMC Bioinformatics. 2007; 8:83–95. 17346331 10.1186/1471-2105-8-83 PMC1838432

[6] Fischer S., Brunk B.P., Chen F., Gao X., Harb O.S., Iodice J.B., Shanmugam D., Roos D.S., Stoeckert C.J. Jr Using OrthoMCL to assign proteins to OrthoMCL-DB groups or to cluster proteomes into new ortholog groups. Curr. Protoc. Bioinform. 2011; doi:10.1002/0471250953.bi0612s35. 10.1002/0471250953.bi0612s35 PMC3196566 21901743

[7] Nakaya A., Katayama T., Itoh M., Hiranuka K., Kawashima S., Moriya Y., Okuda S., Tanaka M., Tokimatsu T., Yamanishi Y. KEGG OC: a large-scale automatic construction of taxonomy-based ortholog clusters. Nucleic Acids Res. 2013; 41:D353–D357. 23193276 10.1093/nar/gks1239 PMC3531156

[8] Huerta-Cepas J., Capella-Gutierrez S., Pryszcz L.P., Marcet-Houben M., Gabaldon T. PhylomeDB v4: zooming into the plurality of evolutionary histories of a genome. Nucleic Acids Res. 2014; 42:D897–D902. 24275491 10.1093/nar/gkt1177 PMC3964985

[9] Sonnhammer E.L., Ostlund G. InParanoid 8: orthology analysis between 273 proteomes, mostly eukaryotic. Nucleic Acids Res. 2015; 43:D234–D239. 25429972 10.1093/nar/gku1203 PMC4383983

[10] Uchiyama I., Mihara M., Nishide H., Chiba H. MBGD update 2015: microbial genome database for flexible ortholog analysis utilizing a diverse set of genomic data. Nucleic Acids Res. 2015; 43:D270–D276. 25398900 10.1093/nar/gku1152 PMC4383954

[11] Huerta-Cepas J., Szklarczyk D., Forslund K., Cook H., Heller D., Walter M.C., Rattei T., Mende D.R., Sunagawa S., Kuhn M. eggNOG 4.5: a hierarchical orthology framework with improved functional annotations for eukaryotic, prokaryotic and viral sequences. Nucleic Acids Res. 2016; 44:D286–D293. 26582926 10.1093/nar/gkv1248 PMC4702882

[12] Galperin M.Y., Kristensen D.M., Makarova K.S., Wolf Y.I., Koonin E.V. Microbial genome analysis: the COG approach. Brief. Bioinform. 2017; doi:10.1093/bib/bbx117. 10.1093/bib/bbx117 PMC6781585 28968633

[13] Zdobnov E.M., Tegenfeldt F., Kuznetsov D., Waterhouse R.M., Simao F.A., Ioannidis P., Seppey M., Loetscher A., Kriventseva E.V. OrthoDB v9.1: cataloging evolutionary and functional annotations for animal, fungal, plant, archaeal, bacterial and viral orthologs. Nucleic Acids Res. 2017; 45:D744–D749. 27899580 10.1093/nar/gkw1119 PMC5210582

[14] Altenhoff A.M., Glover N.M., Train C.M., Kaleb K., Warwick Vesztrocy A., Dylus D., de Farias T.M., Zile K., Stevenson C., Long J. The OMA orthology database in 2018: retrieving evolutionary relationships among all domains of life through richer web and programmatic interfaces. Nucleic Acids Res. 2018; 46:D477–D485. 29106550 10.1093/nar/gkx1019 PMC5753216

[15] Kriventseva E.V., Tegenfeldt F., Petty T.J., Waterhouse R.M., Simao F.A., Pozdnyakov I.A., Ioannidis P., Zdobnov E.M. OrthoDB v8: update of the hierarchical catalog of orthologs and the underlying free software. Nucleic Acids Res. 2015; 43:D250–D256. 25428351 10.1093/nar/gku1220 PMC4383991

[16] Trachana K., Larsson T.A., Powell S., Chen W.H., Doerks T., Muller J., Bork P. Orthology prediction methods: a quality assessment using curated protein families. Bioessays. 2011; 33:769–780. 21853451 10.1002/bies.201100062 PMC3193375

[17] Waterhouse R.M., Kriventseva E.V., Meister S., Xi Z., Alvarez K.S., Bartholomay L.C., Barillas-Mury C., Bian G., Blandin S., Christensen B.M. Evolutionary dynamics of immune-related genes and pathways in disease-vector mosquitoes. Science. 2007; 316:1738–1743. 17588928 10.1126/science.1139862 PMC2042107

[18] Bovine Genome S., Analysis C., Elsik C.G., Tellam R.L., Worley K.C., Gibbs R.A., Muzny D.M., Weinstock G.M., Adelson D.L., Eichler E.E. The genome sequence of taurine cattle: a window to ruminant biology and evolution. Science. 2009; 324:522–528. 19390049 10.1126/science.1169588 PMC2943200

[19] Hoy M.A., Waterhouse R.M., Wu K., Estep A.S., Ioannidis P., Palmer W.J., Pomerantz A.F., Simao F.A., Thomas J., Jiggins F.M. Genome sequencing of the phytoseiid predatory mite metaseiulus occidentalis reveals completely atomized hox genes and superdynamic intron evolution. Genome Biol. Evol. 2016; 8:1762–1775. 26951779 10.1093/gbe/evw048 PMC4943173

[20] i, K.C. The i5K Initiative: advancing arthropod genomics for knowledge, human health, agriculture, and the environment. J. Hered. 2013; 104:595–600. 23940263 10.1093/jhered/est050 PMC4046820

[21] Kriventseva E.V., Rahman N., Espinosa O., Zdobnov E.M. OrthoDB: the hierarchical catalog of eukaryotic orthologs. Nucleic Acids Res. 2008; 36:D271–D275. 17947323 10.1093/nar/gkm845 PMC2238902

[22] Waterhouse R.M., Zdobnov E.M., Kriventseva E.V. Correlating traits of gene retention, sequence divergence, duplicability and essentiality in vertebrates, arthropods, and fungi. Genome Biol. Evol. 2011; 3:75–86. 21148284 10.1093/gbe/evq083 PMC3030422

[23] Waterhouse R.M., Seppey M., Simao F.A., Manni M., Ioannidis P., Klioutchnikov G., Kriventseva E.V., Zdobnov E.M. BUSCO applications from quality assessments to gene prediction and phylogenomics. Mol. Biol. Evol. 2017; 35:543–548. 10.1093/molbev/msx319 PMC5850278 29220515

[24] Ondov B.D., Treangen T.J., Melsted P., Mallonee A.B., Bergman N.H., Koren S., Phillippy A.M. Mash: fast genome and metagenome distance estimation using MinHash. Genome Biol. 2016; 17:132–146. 27323842 10.1186/s13059-016-0997-x PMC4915045

[25] Simao F.A., Waterhouse R.M., Ioannidis P., Kriventseva E.V., Zdobnov E.M. BUSCO: assessing genome assembly and annotation completeness with single-copy orthologs. Bioinformatics. 2015; 31:3210–3212. 26059717 10.1093/bioinformatics/btv351

[26] Federhen S. The NCBI Taxonomy database. Nucleic Acids Res. 2012; 40:D136–D143. 22139910 10.1093/nar/gkr1178 PMC3245000

[27] Steinegger M., Soding J. MMseqs2 enables sensitive protein sequence searching for the analysis of massive data sets. Nat. Biotechnol. 2017; 35:1026–1028. 29035372 10.1038/nbt.3988

